# Performance of castor oil polyurethane resin in composite with the piassava fibers residue from the Amazon

**DOI:** 10.1038/s41598-024-54000-4

**Published:** 2024-03-20

**Authors:** Rosinaldo Rabelo Aparício, Gabrielle Machado dos Santos, Viviane Siqueira Magalhães Rebelo, Virgínia Mansanares Giacon, Cristina Gomes da Silva

**Affiliations:** Programa de Pós-Graduação Em Ciência E Engenharia de Materiais, Universidade Federal Do Amazonas, Manaus, Brazil

**Keywords:** Composite boards, Mercerized piassaba fibers, Castor oil-based resin, Engineering materials, Materials science, Biomaterials, Structural materials

## Abstract

The use of castor oil in producing polyurethane resins has been identified as one of the most promising options for the industry. The piassava fibers waste generated by the industry on a large scale presents excellent properties as a reinforcing agent due to its high lignin content characterized by chemical tests and FTIR. Composite boards consisting of a higher content of mercerized piassava fibers (10 mm, 85 wt.%) reinforced polyurethane castor oil-based resin (prepolymer (PP) and polyol (OM)) exhibited excellent performance. Composites with these properties have strong potential for medium-density applications ranging from biomedical prosthetics to civil partition walls and insulation linings. Alkali treatment removed the superficial impurities of piassava fibers, activating polar groups, and physical characterization reported excellent performance for all composites. Among the composites, the CP3 sample (composite reinforced with piassava fibers (85 wt.% fibers; 1.2:1—PP:OM)) stood out with higher density and lower swelling and water absorption percentage than other composites. FTIR results indicated NCO traces after the resin cured in the PU3 (1.2:1—PP:OM), possibly contributing to the interaction with the fibers. DMA results reported relevant information about more flexibility to CP1 (composite reinforced with piassava fibers (85 wt.% fibers; 0.8:1—PP:OM)) and CP3 than CP2 (composite reinforced with piassava fibers (85 wt.% fibers; 1:1—PP:OM)). The results suggest that the proper combination with natural products must lead to composites with potential applications as engineering materials.

## Introduction

Composites based on partially renewable materials reinforced with lignocellulosic fibers are an excellent economic, social, and environmental improvement option^1,2^. The raw material composites properties make it possible to obtain composites with low density, non-toxicity, low cost, biodegradability, and good thermal and mechanical properties^3,4^.

The research constantly advances the application of diversified residues of lignocellulosic fibers as reinforcement and replacing the polymeric matrix, such as rice husk^5^, banana stem^6^, pine nut husk^7^, and sugar cane straw^8^. The "green" polyurethane composed of vegetable oils was initially developed by university researchers and recently applied by the industry following the tendency. A polyol derivate of vegetable oils, especially from the castor plant, have been applied, such as adhesives^9^, blends^10^, thermal insulation and composites green planners, and others, initially between researchers at the university and recently by the industry. Polyurethane adhesives have been developed from natural oils. More recently, Kraft lignin^11,12^ offers performance equivalent to petroleum derivatives and has low cost, as reported in the literature^13^.

Among various vegetable fiber sources available, some industry segments in Brazil use piassaba fibers from the Amazon region (*Leopoldinia piassaba*). Researchers recently incorporated the piassaba residues into composites to study their main properties^14^. In a short time, industries and consumers have already accepted products prepared with vegetable fibers. As well as other vegetable fibers, the piassaba presents attractive advantages, such as low cost, low density, excellent mechanical properties, recyclability, and biodegradation^15^. In Brazil, Amazon piassaba fibers are used in handicrafts and constructions, and their more popular product is brooms. The waste from brooms' production is the primary residue applied in this work, and they have presented good results, already reported in the literature^14,15^.

The proper blend with polyurethane derived from the castor bean and a high percentage of vegetable fibers has shown promising results in recent years. Almeida et al.^16^ evaluated the mechanical properties of perpendicular traction (1.68 MPa), static bending (15.2 MPa), elasticity modulus (2466 MPa), and pullout strength index on the composite surface (1392 MPa). Adopting wood-bamboo proportion variations and a mass ratio of 90% of particles and 10% castor polyurethane resin, the results were superior to those presented by Standards^17,18^ indicating that composites produced with high amounts of lignocellulosic reinforcement present quality that meets regulatory standards.

Mesquita et al.^2^ used *açaí* fiber residues mercerized with NaOH to produce the composite with a mass percentage of 15% castor polyurethane resin. They obtained physical–mechanical results that suggest a potential for production and consequent commercial use in civil construction and furniture. Sugahara et al.^19^ compared the mechanical properties of composites using castor polyurethane and urea–formaldehyde. Sugarcane bagasse particles and eucalyptus residues reinforced the composites in the proportion of 90% (m/m) of fibers and residue and 10% (m/m). The blend of the fibers showed greater efficiency in physical and mechanical properties for international standards. Nasser et al.^20^ evaluated the mechanical properties, flexural strength for the rupture module, elasticity module, sheer tensile strength, and tensile strength of the composites. Polyurethane resin (10%, w/w) of castor bean impregnated with the peanut shells and bamboo residues prepared the composites and had satisfactory physical–mechanical performance, according to the standards^17,18^.

Faria et al.^1^ prepared castor polyurethane resin and coconut fiber composites (30 to 60 wt.%). The authors evaluated the water absorption by SEM after 20 days of immersion, apparent density, Izod impact, tensile, and fiber/matrix interface. The authors concluded that the composites with 60% fibers could be applied without prejudice to properties, making them lighter and cheaper. Thus, composites with a high proportion of fibers and castor polyurethane resin are an alternative for obtaining environmentally friendly and easily obtainable materials.

This study focused on preparing and characterizing the composites based on a "green" polyurethane reinforced with a high percentage (85 wt.%) of short piassaba fibers residue from the industry. The fibers were previously submitted to the alkaline treatment (10% NaOH) to remove the surface impurities and fatty acids, exposing the main surface polar groups. Furthermore, the different proportions concerning prepolymer and polyol (castor oil) were studied before preparing the composites. When conducting an evaluation, it was observed that natural products yielded composites with highly promising mechanical, thermal, morphological, and physicochemical results. This finding indicates a significant potential for developing and producing composites that incorporate a high content of natural products.

## Experimental section

### Materials and methods

PU resin was kindly donated by Plural Química, São Carlos/SP, Brazil. This PU resin is a bicomponent based on LECOPOL E 0921 (Prepolymer) and LECOPOL F0911 (polyol). The polyol was synthesized from castor oil, with a specific mass of 1.0 to 1.2 g cm^−3^. The prepolymer was synthesized from diphenylmethane diisocyanate (MDI) and pre-polymerized with polyol based on castor oil, with a specific mass of 1.24 g cm^−3^. Piassava fiber waste was donated by Amazon Limpa in Manaus/AM, Brazil.

### Mercerization

The fibers were cut using scissors (around 10 mm) and were immense in the 10 wt.% NaOH solution for a period of 1 h at room temperature. Then, they were washed until pH = 7 ± 1 and dried at room temperature. The fibers were dried in an oven at 105 °C for 24 h before use.

### Prepolymer and polyol characterization for determining the equivalent weight of polyurethane

The equivalent weight of prepolymer (PP) and polyol (OM) was determined through the percentages of free isocyanates in the prepolymer (following the ASTM 2572 Standard) and the hydroxyl index of the polyol derived from castor oil (following the ASTM D 4274 Standard). After determining the exact proportion of the equivalent weight of the polyurethane, the proportions between prepolymer and polyol studied in this article were determined, as shown in Table 1 (the prepolymer (PP) and polyol (OM) proportions).

**Table 1:** Molar proportions prepolymer (PP) and polyol (OM) to polyurethane resins (PU) and composites (CP) reinforced with piassava-treated fibers (85 wt.%).

Composites	Resin	Molar Proportion (PP:OM)
CP1	PU1	0.8: 1.0
CP2	PU2	1.0:1.0
CP3	PU3	1.2:1.0

The ratio PP:OM was varied, and the curing process was flowed by FTIR analysis. The cured resin, followed by the intensity peak NCO (2280 cm^−1^) from FTIR bands, was consumed according to the polyol. This event was obtained from 0.8 g to 1.2 g (prepolymer, PP), maintaining the OM constant at 2.5 g. Then, three different compositions were chosen for this study: 0.8 g, 1.0 g, and 1.2 g PP to 2.5 g OM.

The treated piassava fibers (85 wt.%) and resin were manually mixed for 15 min, distributed in a metal mold (400 × 400 × 10 mm), and formed by thermo-pressing under 15 MPa/100 °C/10 min.

### Characterizations

#### Physicochemical characterization of piassava fiber

Piassava fibers compositions without treatment and treated with 10 wt.% NaOH solution was characterized according to TAPPI Standards. TAPPI T204 cm-97 determined the extractive content and TAPPI T222m-88 determined Klason lignin content. Holocellulose content was determined according to TAPPI T19m54. The α-cellulose content was determined by T429 cm-10 adapted. Ash content was measured using the TAPPI 413 om-02.

#### Physical and mechanical characterization of the composites

The density, moisture content, swelling, and flexural resistance tests were performed according to the Brazilian Association of Technical Standards (NBR 14,810–2). The water absorption test was carried out following standard NBR14810-3.

#### X-ray fluorescence (FRX) of prepolymer and polyol

The prepolymer and polyol ray fluorescence spectrometry was performed on the Epsilon 3XL model from the Malvern Panalytical brand, using 2 mL of samples in plastic cuvettes with 3.6 μm polyester film.

#### Thermogravimetry (TGA) and differential scanning calorimetry (DSC)

Thermal analyses of piassava fibers, prepolymer, polyol, polyurethanes, and composites were performed on SDT (Q600, TA Instruments). The measurements were carried out from room temperature to 700 °C and a heating rate of 10 °C min^–1^ under a nitrogen atmosphere (30 ml min^−1^ flow).

### Infrared spectroscopy (FTIR)

Fourier transform infrared spectra were obtained from a Spectrum IRAffinity-1S- Shimadzu Spectrometer with a spectral resolution of 4 cm^−1^. For piassava fibers, prepolymer, polyol, polyurethanes, and composites, the spectrum was obtained from the horizontal attenuated total reflectance (HATR) technique using a Germanium crystal. The spectra in the region 500–4000 cm^−1^ were run, and the averaged spectrum was plotted as a percent transmittance curve versus wavenumbers with 16 scans.

#### Thermodynamic mechanical analysis (DMA)

The thermodynamic mechanical analysis (DMA) was carried out on a thermal analyzer (Q800, TA Instruments) equipped with a flexural clamp film in multifrequency mode. The dimension samples were 64 mm × 12 mm × 3.2 mm, following the parameters: oscillation amplitude of 20 mm, frequency of 1 Hz, temperature range from -130 °C to 200 °C, and heating rate of 2 °C min^−1^.

### Scanning electron microscopy (SEM)

Scanning electron microscopy (SEM) was carried out on Quanta FEG-250 equipment with the Large Field vacuum SED (LFD) detector in auto vacuum conditions. The electron acceleration voltage used was 5 kV. The samples were dried in the oven (105 °C) air circulation for 4 h, then coated with a thin gold layer (20 nm) using Sputtering Leica EM ACE-200 equipment.

## Results and discussion

### Polyurethane characterizations

#### FTIR

**Figure 1:**
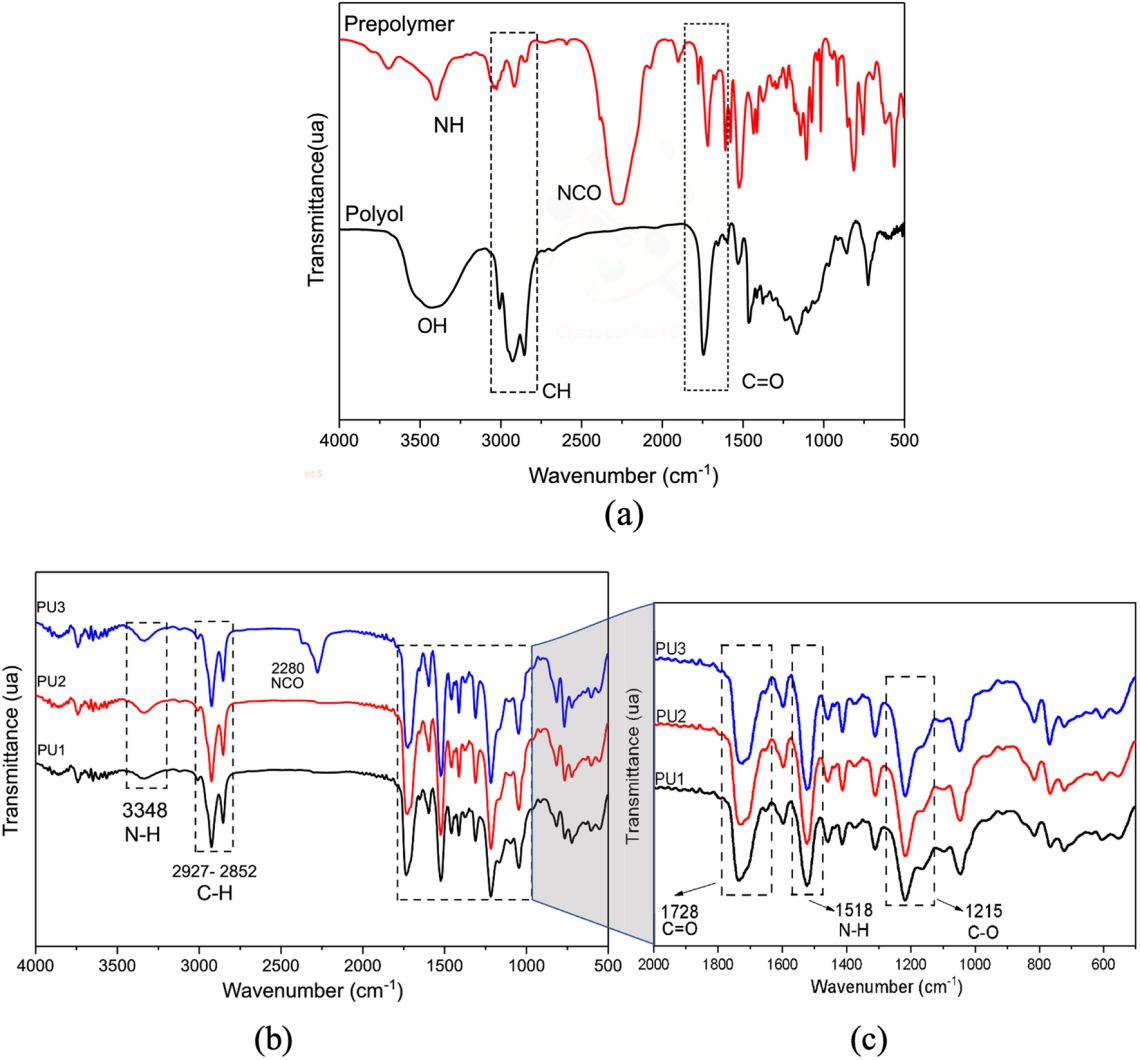
FTIR spectrum of (**a**) the prepolymer and polyol groups; (**b**) PU1, PU2, and PU3 resins after polymerization (72 h) prepolymer/polyol; (**c**) Spectrum resins from 2000 to 500 cm^−1^ referents to C=O, N–H, and C-O absorption bands.

FTIR characterized the polyol, prepolymer, and PU1, PU2, and PU3 resins in the 400–4000 cm^−1^ range, as shown in Fig. 1. Figure 1a shows the main bands to prepolymer to NH (3348 cm^−1^) and NCO (2280 cm^−1^) were stretching bands^21^. Polyol bands are presented at 1740 cm^−1^ and 3400 cm^−1^, corresponding to carbonyl (C=O: stretches of carbon bonds) and free hydroxyl groups (-OH). Other polyol characteristic bands are located at 2800–3000 cm^−1^ to CH stretching vibrations, CH_2_ (asymmetric: 2920; symmetric: 2869), and CH_3_ (asymmetric: 2972; symmetrical: 2885) and stretching bands^22–24^.

The highlight peaks in the spectrum in Fig. 1b correspond to the bands confirming the polymerization after 72 h, using the proportions previously described. The band at 3337 cm^−1^ is correlated to (NH) stretching vibration bands detected in the prepolymer (Fig. 1a) and PU resins (Fig. 1b). Best seen in Fig. 1c, the main groups identified were NH (amine II: 1518 cm^−1^; δ_N-H_ + υ_C-N_ + υ_C-C_), CN (stretching: 1250 cm^−1^), NH (amine III, IV, V: 1250–770 cm^−1^)^22,25^.

In Fig. 1b, the analysis detected that a trace of the isocyanate group (NCO, at 2280 cm^−1^) was present in the PU3 resin, even after the reaction, indicating that the PP was used in excess. PU1 and PU2 curves did not present this peak, indicating that the isocyanate group was well consumed during the cure reaction (after 72 h). Similar results are observed in the literature when the PP is used excessively to prepare the PU resin and is not completely consumed after the reaction and cure process^9^.

The band attributed to the amide group III is identified by the bands NH at 1302 cm^−1^ and C-O at 1220 cm^−1^, correlated to the C-N and N–H stretch vibrations. Besides, other essential peaks were detected referent to flexible polyol at 1706 cm^−1^ and 1041 cm^−1^ correlated to carbonyl groups (C=O) and aromatic rings at 1593^25,26^.

### Thermal analysis (TGA and DSC)

**Figure 2:**
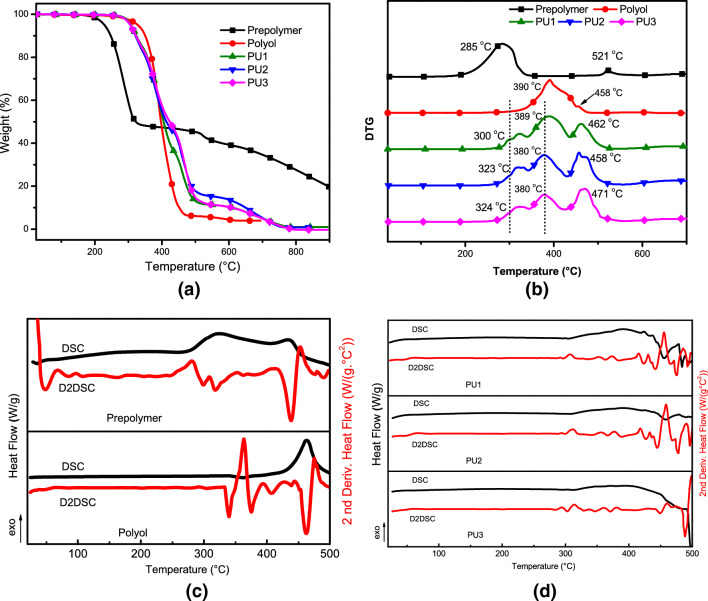
Thermal analysis curves of the prepolymer, polyol, PU1, PU2, and PU3: (**a**) TGA; (**b**) DTG; (**c**, **d**) DSC/D2DSC; under N_2_ atmosphere (30 mL.min^−1^) and heating rate 10 °C min^−1^.

Figure 2 presents the TGA/DTG, DSC curve, and D2DSC (the second derivative of the DSC signals) recorded of the samples analyzed. The prepolymer curve is shown in Fig. 2a, b, which presents two well-defined stages: firstly, the fatty acids' decomposition at 120 °C—370 °C (about 52% loss mass). The prepolymer curve DSC/ D2DSC (Fig. 2c) confirmed the TGA/DTG events by exothermic peaks at 328 °C and 500 °C. The polyol DTG curve reports a peak at 390 °C (94% loss mass) correlated with ester bond decompositions, dehydrogenation, hydroxyl degradation, and alkyl groups polycondensation. All groups are present in the fatty acid monoglyceride structure.

Previous studies reported in the literature proposed that, in general, all polyurethanes reveal three main degradation steps^27–29^. In the first step (Fig. 2b and Fig. 2d), at around 320 °C the urethane bonds degraded (polyurethane dissociation in isocyanates and alcohol molecules), and the loss mass of the free MDI (diisocyanate) by evaporation. Consequently, in this range, primary and secondary amines and olefin were formed^21,28,29^. In the second event, around 350–450 °C, high energy may correspond to CO degradation, soft polyurethane segments, and single or double bands, as C=O, C=C, C–O, C–O–H^29^. The third degradation stage occurred in the same range of temperature, between 400 and 500 °C (43% loss mass), as displayed by D2DSC (Fig. 2d) correlating to ester bond decompositions presented in the prepolymer structure, besides flexible regions and functional groups (N–H and C=O)^7,8^.

### X-ray fluorescence spectroscopy results

**Figure 3:**
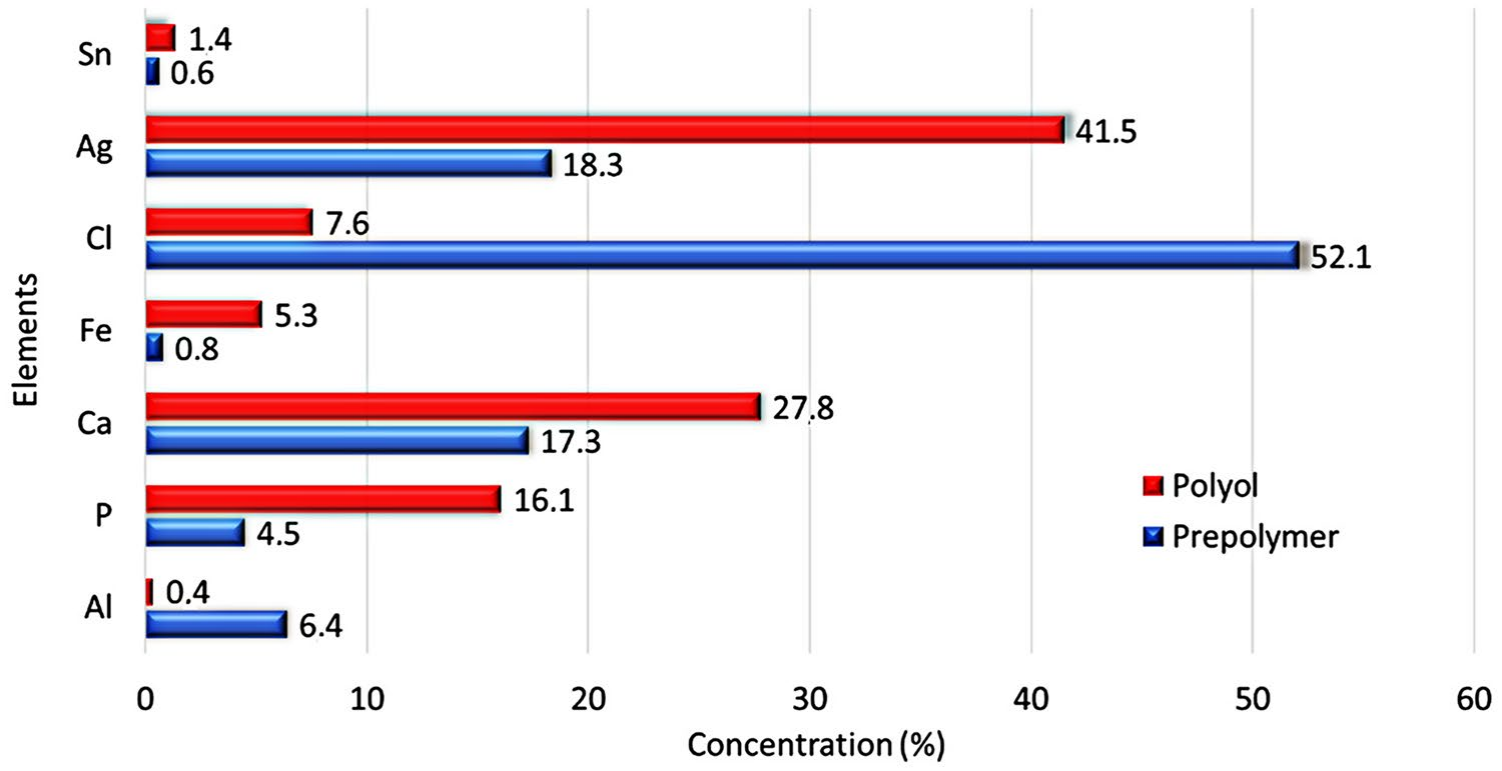
X-ray fluorescence spectroscopy results of the prepolymer and polyol, from Sodium to Uranium elements.

The results reported by X-ray fluorescence spectroscopy confirmed traces of the inorganic contaminants in the prepolymer and polyol. These contaminants probably were from raw materials used during the industrial preparation. The percentage found of the chemical elements in both materials is listed in Fig. 3. Chloride (Cl, 52.1%) and silver (Ag, 41.5%) were the major inorganic components found in these raw materials by manufacturers (listed in the datasheet).

Chloride forming the inorganic chloride salt, probably from the acid chloride residual used in the prepolymer reaction, is regularly used to adjust the pH. The silver detected, which may be linked to oxygen, is commonly incorporated as a flame retardant. Calcium (Ca, 27.8%) and phosphate (P, 16.1%) detected in relevant percentages are commonly used as an inorganic load. All inorganic compounds are responsible for the residual raw material TGA analysis indicates (Fig. 2). The results of the composites' properties can be influenced by the components found in the raw materials.

### Piassava fibers characterizations

#### Chemical characterization

The treatment of natural fibers by sodium hydroxide (NaOH) removes a portion of extractives, hemicelluloses, lignin, pectin, wax, and oil-covering materials, making the fiber surface clean^14,30^. Table 2 shows the average values of fibers' chemical composition (lignin, cellulose, hemicellulose, extractives, and ashes) untreated and treated with NaOH (10%) and the significant reduction (p-value < 0.05) of the same components after the treatment.

**Table 2:** Chemical composition of Piassava fiber untreated and treated (10% NaOH).

Composition	Untreated (wt%)	Treated (wt%)
Extractives	8.9 ± 0.3	4.8 ± 0.1
Klason lignin	34.9 ± 0.4	31.2 ± 0.2
Holocellulose	41.9 ± 1.0	35.2 ± 0.3
Alfa Cellulose	25.2 ± 0.5	23.8 ± 0.1
Hemicellulose	16.7 ± 1.0	11.4 ± 0.3
Ash	1.1 ± 0.1	6.2 ± 0.3
Moisture	9.6 ± 0.3	14.5 ± 0.2

The insoluble lignin values ranged from 34.9% to 31.2% for untreated fiber and treated, respectively. The mercerization process reduces lignin, natural oils covering, and fiber aggregation. The study reported by d'Almeida et al.^31^ with piassaba *Attalea funifera* without treatment presented lignin and cellulose values higher than those obtained in this study, as 48.4% and 31.6%, respectively. The α-cellulose values ranged from 25.2% to 23.6% for untreated fiber and treated, respectively. These results were lower than the piassava *Attalea funifera*, around 31.6% to natural fiber^31^. The difference between fibers' composition could be due to species, soils, and climate differences affecting these values^31–33^.

A study with treated sugarcane bagasse (40 wt % NaOH) showed a significant reduction in hemicellulose and lignin content due to the mercerization process. The dissolution of these components (amorphous) is faster than cellulose crystals; however, the ash content remained constant^34^. In this study, the ash content of the treated fibers (6.2%) was higher than without treatment (1.1%). This increase is probably due to elements present, such as sodium; as described by^14^, the inorganic percentage after treatment (NaOH 10%) increased by around 23%^14^. Also, moisture is enhanced after the treatment, promoting a higher swelling due to the sodium (Na +) present in the structure (Na–cellulose-I lattice), increasing distances between the cellulose chain and consequently filling the space with water molecules^35^. In general, the increase of the moisture content after the alkaline process may be correlated with removing protective waxes or hydrophobic components (from ~ 8 to ~ 4%), exposing the fiber's surface to make it more hydrophilic^36–38^. These characteristics contribute to a decrease in the mechanical properties of the composites^39^. On the other hand, reducing extractives promotes a better adhesion between reinforcement and matrix, as it favors the increase of the surface roughness of the fiber1, consequently increasing the mechanical properties^33^.

**Figure 4:**
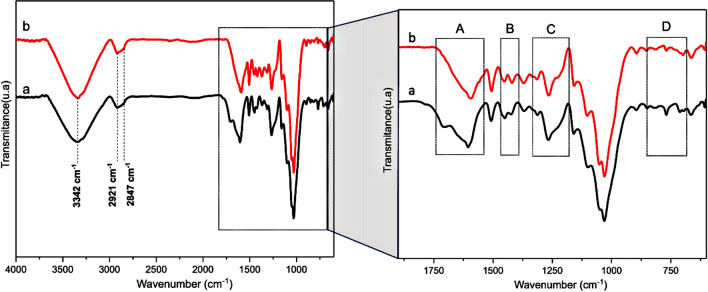
Infrared spectra of the piassava fibers (**a**) untreated and (**b**) treated with NaOH 10%.

Figure 4 highlights the main bands of the fiber structure analyzed by FTIR. Figure 4a shows that the intensity band at 3342 cm^−1^ corresponds to the stretching or axial deformation of the hydroxyl groups (OH) bound to the cellulose structure^40^. The bands detected around 2921 cm^−1^ and 2847 cm^−1^ for both samples are correlated with CH stretching of the methylene (CH_2_) and methyl (CH_3_) groups from polysaccharides compound^41^.

Figure 4b highlights four relevant regions in the FTIR spectra (A, B, C, and D). In the 1700 to 1600 cm^−1^ range, the C=O stretching bond from carboxyl and acetyl ester groups is present in the hemicellulose structure. The hemicellulose peak at 1705 cm^−1^ was not detected in the untreated fiber spectrum^42–44^, indicating their relevant remotion promoted by the alkali treatment, as reported in Table 2.

Between 1500 cm^−1^ and 1000 cm^−1^, typical aromatic bands (C–C, C=C, C-O, C–O–C, CH_n_, and C-H) correlate to aromatic rings were detected, mainly from the lignin structure^45^. The bands attenuated concerning untreated fibers are indicative of the lignin extracted. The band at 1050 cm^−1^ indicated the presence of the C–O–C, C=C, and C–C-O groups from cellulose, hemicellulose, and lignin structures^45^. Bands at 1452 and 1426 cm^−1^ are associated with CH_2_ and C-H, respectively, and –COO at 1262 cm^−1^ is present in the hemicellulose structure^40^.

Besides, the band at 1158 cm^−1^ is referent to C–O–C cellulose and hemicellulose groups and C-O from the cellulose group (1023 cm^−1^)^46^. Other bands between 900 to 600 cm^−1^ were detected and can be attributed to glycosidic and C–OH groups^47,48^.

Merlini et al.^49^ reported a study about banana fibers using a similar treatment applying NaOH 10% in different periods (1 to 8 h). The band at 1726 cm^−1^ showed the gradual reduction associated with the treatment, partially reducing the hemicellulose and modifying the fiber chemical structure^49^. Similar results were reported by^50,51^, who observed the reduction of the C-H and C=O stretching band (1712 cm^−1^) after applying the soda treatment previously presented in the hemicellulose structure^50^.

### Thermal analysis (TGA and DSC)

**Figure 5:**
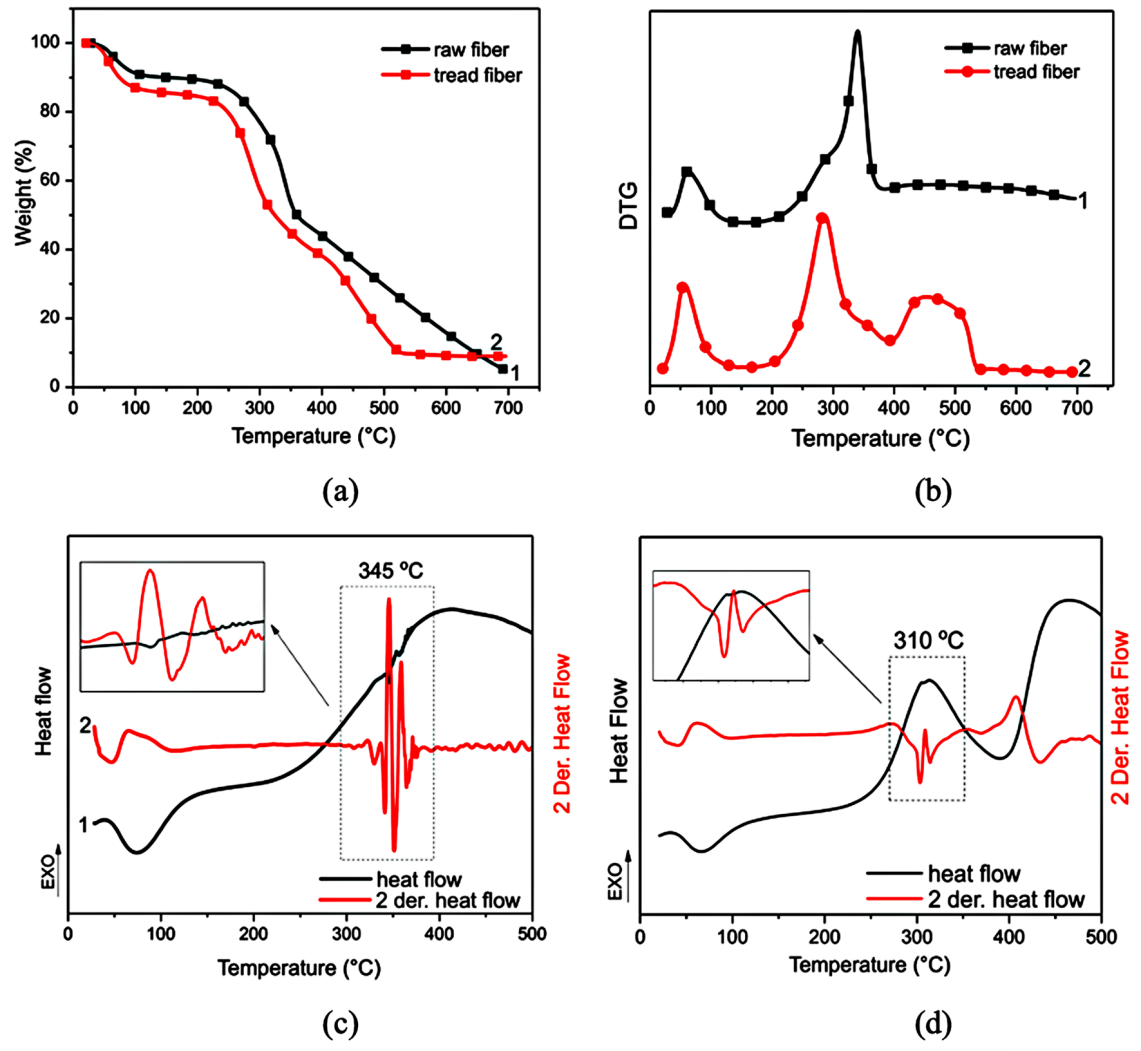
TGA/DTG curves of (**a**) untreated piassaba fibers (UPF) and (**b**) treated piassaba fibers (TPF); N_2_ atmosphere (30 mL.min^−1^) and the rate of 10 °C min^−1^.

Thermal analysis characterized the untreated piassaba fibers (UPF) and treated (TPF), as shown in Fig. 5a (TGA) and Fig. 5b (DTG), reporting the main peaks correlated to intramolecular water and most compounds in their structure (cellulose, lignin, and hemicellulose). The molecular water evaporating detected the first peak, around 60 °C, in both fibers. The loss mass in this step was 10% (PFU) and 14.5% (PFT) concerning the fibers' moisture, as previously listed in Table 2.

UPF presented a shoulder at 285 °C, probable to the hemicellulose decomposition, followed by an intense peak at 339 °C correlated to α-cellulose decomposition. In the range of 400 °C to 700 °C, the analysis presented a loss mass of around 39%, probably due to the lignin decomposition and other inorganic components. This value concerns the chemical characterization reported in Table 2.

After the treatment, a percentage of the components were removed, promoting a shift to a lower temperature of the cellulose degradation (284 °C), probably overlapping the hemicellulose peak. Besides, the cellulose and other structures are exposed after alkaline treatment, influencing the degradation temperature.

A small shoulder observed at 360 °C may correspond to the cellulose structures with high density. The treatment promoted the exposure of the fiber structures; consequently, a large peak was detected after 400 °C by changing the baseline (Fig. 5b), corresponding to a 28% loss of mass. Probably, the event from 390 °C was about the lignin degradation involving the hydroxyl groups present in *p-*hydroxyphenyl (H), guaiacyl (G), and syringyl (S) structures^52,53^. This process promotes breaking the functional groups (ether groups), lignin degradations, and phenol group formation^52,54^. According to Table 1 results, the ash content residues were between 2.8% and 9.0%, reporting similar TGA results in the literature.

D'Almeida et al.^31^ identified the main endothermic events in the thermal analysis from piassaba fibers; the first peak at 74 °C concerning humidity at 276 °C was associated with hemicellulose decompositions, and at 345 °C associated with the α-cellulose decomposition. Rebelo et al.^14^ reported results about the nature and treated (NaOH 10%) piassava Amazon fibers associated with moisture, hemicellulose, and α-cellulose decompositions. Furthermore, the authors reported the hemicellulose reduction at 250 °C, associated with the alkaline treatment.

In the DSC curves (Fig. 5), an endothermic peak was detected at 74 °C (raw fiber) and 66 °C (treated fiber) for both fibers, correlated to the moisture present in the superficial structure fibers^55^. Next, an endothermic peak about the cellulose degradation at 346 °C, followed by the cellulose's residual polymerization (indicated by the exothermic peak at 356 °C). In the last step of this analysis, a large exothermic peak at high temperature (up to 400 °C) correlates to the lignin degradation and carbonaceous residue^56,57^

Figure 5 shows the derivative second of the DSC curve (D2DSC or 2DerDSC), detecting the subtle changes in the results. An exothermic event around 310 °C was probably correlated to the hemicellulose degradation, simultaneous to the other component degradations. Endo and exothermic peaks, between 345 °C and 350 °C, may be correlated to cellulose oxidation, decarboxylation, or partial lignin degradation at low temperatures^3,54,58^. Ray et al.^59^ studied the effect of the alkaline treatment in different periods (2 to 8 h) in the jute fibers by DSC. The cellulose peak from endothermic to exothermic modifies the chemical bonds between the constituents^56,60^.

### Composite characterizations

#### Physical and mechanical characterization

Physical and mechanical results are presented in Table 3, and the reference values for the tests adopted by ABNT NBR 14,810–2^17^. Statistically, performing the ANOVA (Analysis of Variance) test for one factor, considering the prepolymer variation as the response factor, it is observed that there was no significant difference between the density values (0.205 < 0.05/F = 1, 68).

**Table 3:** Results of physical–mechanical testing of composites. (*) Note: Means followed by the same letter do not present statistically significant variation for the Tukey test (p > 0.05).

Characterizations	CP1	CP2	CP3	Reference (minimum
Density (Kg/m^3^)	777 ± 21 (a)	799 ± 17(a)	819 ± 21 (a)	**–**
Water absorption (%)	105 ± 7 (a)	71 ± 12 (a)	65 ± 4 (a)	**–**
Swelling* (%)	34 ± 3 (a)	18 ± 3 (b)	19 ± 3 (b)	18
Moisture (%)	13 ± 1 (a)	13 ± 1 (a)	13 ± 1 (a)	4–13%
MOR* (MPa)	7.9 ± 1.1 (a)	14.5 ± 4.0 (b)	13.5 ± 1.5 (b)	11
Thickness (mm)	> 6.0 a 13	> 6.0 a 13	> 6.0 a 13	

**Figure 6:**
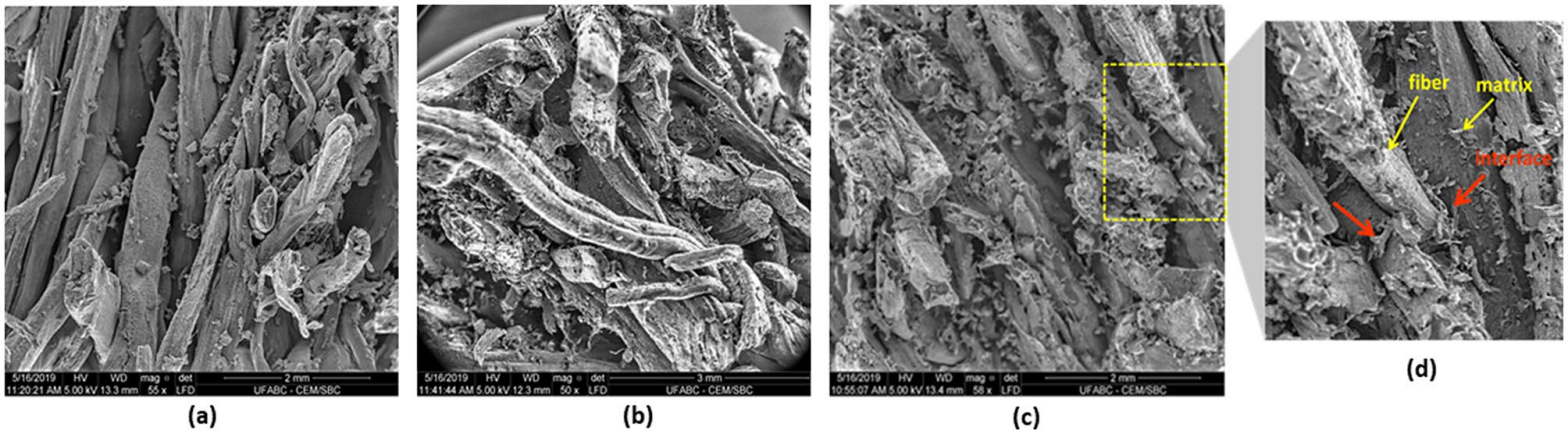
SEM images of the composites' fractured surface (**a**) CP1, (**b**) CP2, (**c**, **d**) CP3.

CP1 composite presented a lower density value than other composites, even with relative moisture values (approximately 13%). Therefore, as expected, CP1 showed higher values in the swelling and water absorption tests after 24 h, (34 ± 3) % and (105 ± 7) %, respectively. These results may be correlated to the PP:MO variations applied in this study, reflecting the degree of crosslinking between the polymeric chains, the residual fraction of the NCO group, or the presence of free polar groups. In addition, other factors, such as the amount of resin retained by the fibers because of the molding process, contribute to the difference in density values obtained. The lower the amount of resin retained in the sample, the lower the composite density. These conditions may have influenced the poor adhesion of the fibers/matrix in the interface region, as shown in the SEM images (Fig. 6). This characteristic contributes to the accommodation of the water molecules into the voids, pores, and cracks at the composite bulk. Furthermore, the water absorption percentage by the fibers is a consequence of the poorly coated polymeric matrix.

Although the composites presented variations in the swelling and water absorption percentage, the moisture values remained constant. It is important to remember that for these materials, parameters related to the presence of water (moisture, water absorption, and swelling) are relevant to their applications^61^.

In general, the results presented (Tabla 3) by composites are within the limit established by the standard. Statistical analysis showed a significant difference between composite results (0.000 < 0.05/F = 15.40), demonstrating that all their analyzed properties are correlated to the prepolymer type applied. Evaluating Tukey's 95% confidence level test was applied to verify which samples are distinct, showing that samples CP2 and CP3 are statistically equivalent and sample CP1 shows a difference between the other samples. Therefore, according to the Brazilian standard, the values obtained for modulus of strength in the bending test are satisfactory and confirm the influence of the prepolymer on the composites' general properties. Evaluating Tukey's 95% confidence level test was applied to verify which samples are distinct, showing that samples CP2 and CP3 are statistically equivalent and sample CP1 shows a difference between the other samples. Therefore, according to the Brazilian standard, the values obtained for modulus of strength in the bending test are satisfactory and confirm the influence of the prepolymer on the composites' general properties.

According to NBR 15,316–2 (2015)^62^, the samples' moisture range met the Brazilian standard's specifications. Statistically analyzing the data, there was no significant change between the prepolymer percentage variations (0.200 < 0.05/F = 1.71). The moisture content is one of the parameters that contributed to the mechanical resistance performance of the panels, which were presented with satisfactory results following the rupture modulus (MOR) test. The CP3 composites were the sample that came closest to the value indicated by the standard. So, statistically analyzing the values shows significant differences in the three variations of prepolymer (0000 < 0.05/F = 15.31).

When composites are prepared based on castor oil (2:1; polyol: prepolymer) reinforced with macadamia seed (20%), the swelling content (2.7%) and water absorption (10.5%) decrease considerably after 24 h^63^, as described by Wechsler et al.^63^. Sánchez et al.^64^ reported plasma-treated bamboo reinforcing composites with 30% castor oil-derived resin, polyol: prepolymer ratio (1.5:1). This resulted in 800 kg/m^3^ density composites, 11.60% water absorption, and 7.50% swelling (24 h). In general, the studies reported by the literature show composite results with higher densities, lower percentages of swelling, and water absorption^65^. These results can be reported to a higher resin percentage and their proportion polyol: prepolymer used. Besides, the amount of material to reinforce the composites should be considered.

These composites developed in this study present themselves as a viable alternative for producing boards with a high content of vegetable fibers, exceptional resistance, and low water absorption. Composite boards such as this are strong contenders for developing sustainable materials in the civil engineering industry, including structured boards for MDF and MDP. Using this material in the applications mentioned could create more sustainable products that align with the circular economy principles and reduce environmental impact. The material has excellent potential for innovative solutions in civil engineering, generating significant interest from academic and industrial stakeholders.

### Scanning electron microscope (SEM)

Figure 6 presents the fracture region of all composites' surfaces after the static flexural test, analyzed by SEM. Different factors, such as fiber-matrix interface, voids, crack propagation, and some preparation problems, influenced the samples. Composites with higher fiber content can result in the lack of resin in some regions, occasionally weakening the sample^15,66^.

A previous report by Rebelo et al. showed the natural piassava fiber SEM images (received from the same source) before and after treatments^14^. The images showed satisfactory recovery of the treated fiber by the resin (Fig. 6a) compared to natural piassava fibers^14^. In addition, it was observed that the fiber distribution was homogeneous throughout the sample despite the high fiber concentration.

Concerning the interface fiber-matrix interface, few regions were observed during the analysis due to the high intensity of the fibers. Figure 6d highlights one of the regions observed in the collected images, as red arrows indicate. The recovery and adhesion are relevant to composites' property and their application.

Few voids were observed in the matrices, which can be attributed to the CO_2_ released after the resin cure process or humidity presented into the structure of the fibers^15,55^. The SEM analysis realized on the composite surface (Figure not shown) revealed an excellent fiber compacted and well-filled by the polymer surface without defects, voids, or cracks, showing a good interaction of the fibers with the polyurethane polymer matrix. This property favors the waterproofing of the composite samples against moisture absorption, one of the main properties applied for the plates^67^.

In general, the regions of the fibers detached from the matrix in the interface regions were commonly observed after the flexural test in the SEM images. The force applied slowly to the sample usually promotes the detachment of the fibers more quickly than the high-impact tests. The fiber/matrix adhesion property is due to free functional groups in the fibers and the matrix^68^. The low adhesion or interaction in the interface region may indicate the low availability of polar groups in the components^69^ or even result from the thin layer of resin applied to the fiber. A large fiber percentage (85 wt. %) to the resin percentage may have been insufficient for the efficient fiber coating (wetting of the fibers) and the filling of the internal channels of the fibers. Enough resin for covering/coating the fibers is a relevant factor that guarantees the excellent adhesion efficiency of the fibers/matrix, better mechanical properties, and the waterproofing/protection of the fibers against moisture. Studies presented in the literature show that the accumulation of water molecules in regions of voids or pores acts as a plasticizer and impairs the mechanical properties of the composite.

### Dynamic mechanical analysis (DMA)

**Figure 7:**
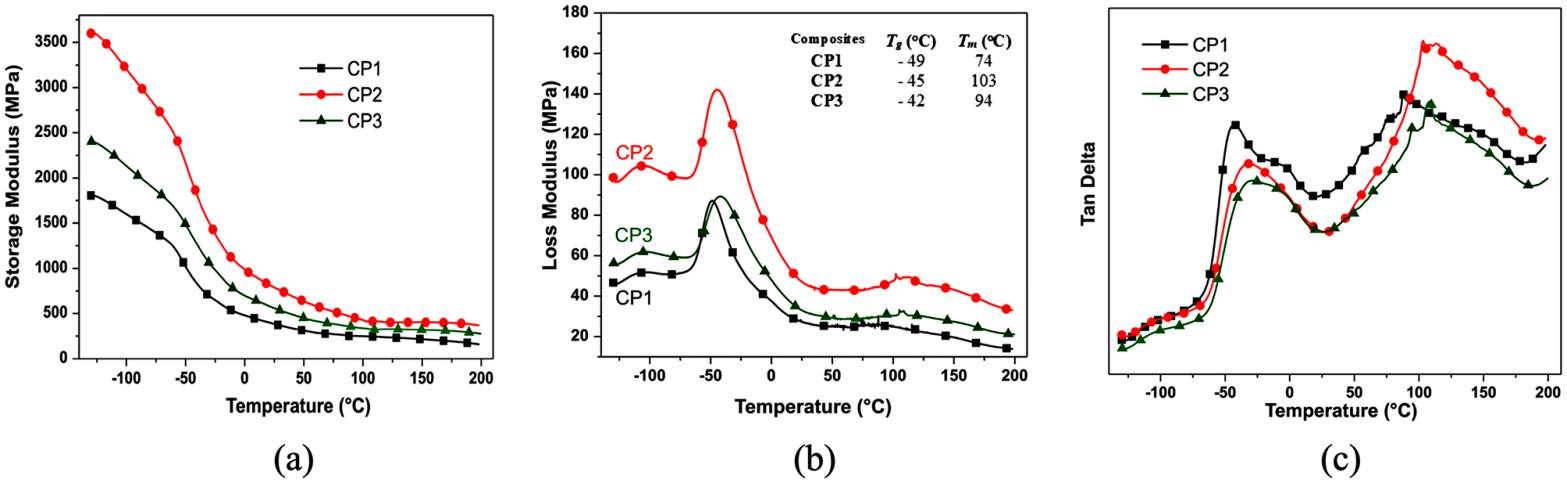
DMA results of the composites CP1, CP2, and CP3: (**a**) Storage Modulus (MPa), (**b**) Loss Modulus (MPa), (**c**) tan δ Modulus.

Reinforced composites were analyzed by DMA, evaluating the performance storage modulus (E'), loss modulus (E"), and tan δ, as seen in Fig. 7. This analysis reported the interaction fibers/matrix in the interface and the interphase region in the temperature range (-130 to 200 °C). Concerning the matrices, this analysis mainly relates to the segments' mobility between crossing points^70^. CP2 exhibited higher E' values than CP1 and CP3 throughout the analysis range, indicating greater material flexibility concerning the others due to the polymers' side groups or terminal groups' movement in its glassy state at low temperatures. In general, the SM (Storage Modulus) values ​​decreased quickly until around 0 °C, the thermal sensitivity of the composites.

The E" curves presented the first peak around − 105 °C, corresponding to the transitions in the polymer's amorphous (non-crystalline) phase, with possible movements or relaxations of side groups and end groups of the chains. In the scanning of the sample, promoted by the temperature increase, there is an abrupt drop in the E' values as a response to the non-crystalline region movements, corresponding to composite glass transition (*T*_*g*_). In Fig. 7b, E" curves showed second and third peaks revealing the *T*_*g*_ and *T*_*m*_ composites temperatures, which vary subtly according to their chemical composition of the matrix.

The excess of reagents (prepolymer or polyol) used in the polymeric resin preparations (PU1 and PU3, respectively) reflected in the growth process of the polymeric chains, the consumption of reagents, and residual small molecules. These parameters are reflected in the *T*_*g*_ and *T*_*m*_ values of all composites.

The loss modulus is directly proportional to the heat dissipated (enthalpy) per cycle and the maximum value of the strain energy during the cycle. This heat dissipation is attributed, for example, to the movement of long segments of the main chain, as occurs in the glass transition, or to relaxations of side segments resulting, such as from rotations around chemical bonds^71^. Tan δ curves express the ability of a material to convert energy, as is the case with polymers in the glass transition region. In this region, relaxations are normally associated with changes in the conformation of groups or segments of the polymer chain resulting from rotations around chemical bonds. The relaxation time measures the mobility of the chains and depends on the molecular structure and temperature, which influences the mobility of the polymer chain and, consequently, the time related to its relaxation^71^. The dependence of the storage and loss modulus on frequency and temperature are represented in the results in Fig. 7. Figure 7a presents the higher E" value (Fig. 7) for the CP2 composite, compared to the others, indicating a movement less flexible of their segments in the material bulk throughout the entire sample scan. At higher temperatures or lower frequencies, the relaxation time of the segments is higher compared to the time scale of the experiment. This is indicative that the material presents more rigid properties, as seen in CP2 results. As the temperature of the experiment increases or the frequency decreases, the storage modulus E" decreases, and the loss modulus presents a maximum, indicating that the material passes from the glassy to the viscoelastic state.

## Conclusions

The industry technology is continuously improved to produce Medium Density Fiberboard according to Brazilian and International Standards. In addition to the automotive and biomedical industries, the civil industry invests heavily in producing fiberboard for furniture, partitions, and coverings for homes, industry, laboratories, hotels, and hospitals, significantly impacting this segment. The proposal of this work allowed a better understanding of the profile of each sample when reinforcing piassaba fibers. The results showed a satisfactory increase in the MOR values of 41.5% when the PP increased from 0.8 to 1.2, while the boards’ density was practically unchanged. Parameters related to the amount of water absorbed by composites are relevant to their application. In this work, the water absorption and swelling percentage decreased by 38.1% and 47%, respectively, maintaining the moisture percentage constant. The images presented by SEM show that the resin covered the fibers but not enough to form a continuous matrix with a good interface region due to a large percentage of fibers in the board (85%). This point can be easily improved by increasing the amount of resin used in the composite preparation, which is sufficient to ensure a more effective coating and improve the matrix-fiber interface. DMA results highlighted the flexibility profile of each composite, indicating the application possibilities, which increased from CP1 and CP3 to CP2. According to the research, the results report the contributions to innovation in composite engineering by using materials from renewable sources, associated with subtle adjustments in the percentage of the resin composition, and a simple treatment of the vegetable fibers residues generated by the industry. In conclusion, the findings of this research underline the importance of exploring the use of renewable resources and effective waste management in the composites engineering industry.

### Supplementary Information

Supplementary Information.

## Data Availability

The manuscript provided the data set generated during the reported study.
