# Supplementary material for: Performance of castor oil polyurethane resin in composite with the piassava fibers residue from the Amazon
Source: Sci Rep. 2024 Mar 20;14:6679. doi: 10.1038/s41598-024-54000-4 (PMC10955110; doi:10.1038/s41598-024-54000-4)
Supplement: Supplementary file 1 — Supplementary Information. [file 41598_2024_54000_MOESM1_ESM.pdf]

Supplementary Information

Performance of castor oil polyurethane resin in composite with the piassava fibers residue from the Amazon

Rosinaldo Rabelo Aparício[1], Gabrielle Machado dos Santos[1], Virgínia Mansanares Giacon[1], Cristina Gomes da Silva[1*]

[1]Universidade Federal do Amazonas, Programa de Pós-Graduação em Ciência e Engenharia de Materiais, Manaus, Brazil

(*) Corresponding author: cristinagomes@ufam.edu.br; cristinaggs@gmail.com

Appendix 1: Determination of equivalent masses of prepolymer and polyol

O determine the equivalent weight (eq. wt) of the prepolymer and polyol, use the values for the percentage of free isocyanates (NCO) in the prepolymer (ASTM, D2772-97 (2010))[1], and the hydroxyl index of the polyol derived from castor oil (ASTM D 4274-16 (2010))[2].

Procedures for determining % free NCO in the prepolymer

To determine the percentage of free NCO, 0.1 g of the prepolymer was dissolved in 25 g of toluene until completely dissolved. Then, 25 mL of the 0.1 N n-dibutyl amine solution was added under stirring for 15 minutes. After that, 100 mL of isopropyl alcohol was added and titrated with a 1.0 mol/L hydrochloric acid solution with bromophenol blue as an indicator. The test was performed in triplicate with a blank sample for reference. The result was calculated using Equation 1 as described.

$$\%NCO = \frac{[(B - V)x\ Nx\ 0{,}0420]}{W}\ x\ 100 \quad (1)$$

B = Volume of hydrochloric acid spent on the blank sample (mL):

V = Volume of hydrochloric acid used with the sample (mL):

N = Exact concentration of the hydrochloric acid solution;

W = Sample (g).

Determination of hydroxyl number in polyol

The methodology employs titration to determine the hydroxyl index. First, 3.7 g of the polyol was dissolved into a flask containing 25 mL of pyridine solution with phthalic anhydride. The system was refluxed for 1 h at 115 ºC ± 2 ºC. After cooling for 30 minutes, 50 mL of

pyridine is added to wash the condenser. The solution is titrated using 0.5 N sodium hydroxide (NaOH) and a phenolphthalein indicator solution (in pyridine 10 g.L-1). The test was carried out in triplicate, and a blank sample was used. Finally, the hydroxyl index was determined using Equation 2.

$$hydroxyl\ index = \frac{(B - A)x\ Nx\ 56.1056}{Ma} \quad (2)$$

B = Volume of NaOH spent on the blank sample (mL):

A = Volume of NaOH spent on the sample (mL):

N = Normality of the NaOH solution;

56.1056 $g.mol^{-1}$ = molar mass of KOH

Ma = Sample (g).

NCO percentage and prepolymer equivalent weight.

Titrations and calculations were carried out using the equation from the standard, determining the free isocyanate percentage present in the prepolymer, with an average result of 25 %. Calculating the weight equivalent to the prepolymer resulted in 168 $g.eq^{-1}$ (Equation 3).

$$Prepolymer\ equivalent\ weight = \frac{42x100}{25} = 168\ g.eq^{-1} \quad (3)$$

The NCO group's weight fraction is 25%, and NCO molar mass is 42 g mol^{-1}.

Hydroxyl Index (OH) and Polyol Equivalent Weight.

Based on Equation 2, the hydroxyl index has an average value of 136 mg KOH/g. The polyol equivalent weight derived from vegetable oil was calculated based on the result obtained, resulting in a value of 412.5 g/eq (as seen in Equation 4).

$$Polyol\ equivalent\ weight = \frac{56.1056x1000}{136} = 412.5 \quad (4)$$

The molar mass of KOH is 56.1056 $g.mol^{-1}$, 1000 mg/g of the sample.

Quantities of polyol and prepolymer for preparing polyurethanes.

For preparation, $6.06x10^{-3}$ g of the prepolymer was used, following a 1:1 molar ratio.

$$\frac{2.5\ g\ de\ polyol\ of\ vegetable\ oil}{412.5\ equivalent\ weight\ of\ vegetable\ oil\ polyol}$$

$$grams\ equivalents\ of\ vegetable\ oil\ polyol = 6.06\ x\ 10^{-3}$$

To comply with the established 1:1 molar ratio, 1.01 g of the prepolymer must react to 2.5 g of polyol.

Reference

[1] American Society for Testing and Materials. ASTM D2572 - 97 Standard Test Methods for Isocyanate Groups in Urethane Materials or Prepolymers Testing, (2010).

[2] American Society for Testing and Materials. ASTM D4274 - 16 Standard Test Methods for Testing Polyurethane Raw Materials: Determination of Hydroxyl Numbers of polyols, (2010).
